# Spatial Dynamics of Dengue Fever in Mainland China, 2019

**DOI:** 10.3390/ijerph18062855

**Published:** 2021-03-11

**Authors:** Yujuan Yue, Xiaobo Liu, Dongsheng Ren, Haixia Wu, Qiyong Liu

**Affiliations:** Chinese Center for Disease Control and Prevention, State Key Laboratory of Infectious Disease Prevention and Control, National Institute for Communicable, Disease Control and Prevention, Beijing 102206, China; yueyujuan@icdc.cn (Y.Y.); liuxiaobo@icdc.cn (X.L.); rendongsheng@icdc.cn (D.R.); wuhaixia@icdc.cn (H.W.)

**Keywords:** spatial characteristics, indigenous dengue cases, domestic imported dengue cases, overseas imported dengue cases

## Abstract

New spatial characteristics of dengue fever in mainland China during 2019 were analyzed. There was a dengue fever outbreak in mainland China in 2019, with 15,187 indigenous cases in 13 provinces, 1281 domestic imported cases from 12 provinces and 5778 overseas imported cases from 47 countries, more than the previous cases during the period 2005–2018, except for in 2014. Indigenous cases occurred in Sichuan, Hubei and Chongqing in 2019. There have been big changes in the spatial distribution and proportion of dengue cases. Indigenous cases were not only located in the southwestern border and southeastern coastal provinces of Yunnan, Guangdong, Guangxi and Fujian but also in the central provinces of Jiangxi and Chongqing. Domestic imported cases were not only from Guangdong, but also from Yunnan. There were five new sources of importation of cases. Overseas imported cases were mainly from Cambodia and Myanmar in 2019. Understanding the new spatial characteristics of dengue fever in China helps to formulate targeted, strategic plans and implement effective public health prevention and control measures.

## 1. Introduction

Dengue fever, mainly transmitted by *Aedes* mosquitoes, is one of the most prevalent mosquito-borne diseases in humans [1]. There are four dengue virus serotypes (DENV 1–4) [2]. Before 1970, only nine countries had experienced severe dengue epidemics. Now, dengue fever is endemic in more than 100 countries in Southeast Asia, the Americas, the Western Pacific, Africa and the Eastern Mediterranean regions, evolving from a sporadic disease to a major public health problem with increasing geographic extension, numbers of cases and disease severity [3]. Dengue is found in tropical and sub-tropical climates worldwide, mostly in urban and semi-urban areas [4]. Despite a risk of dengue infection existing in 129 countries [5], 70% of the actual burden is in Asia [6]. According to an estimation made in 2013, 390 million people had dengue virus infections, and there were 96 million dengue cases annually worldwide (at any level of clinical or sub-clinical severity) [1], more than three times the dengue burden estimate of the World Health Organization (WHO) [7]. The number of dengue cases reported to the WHO has increased by more than eight-fold during the last two decades, from 505,430 cases in 2000 to 4.2 million in 2019. Reported deaths between the years 2000 and 2015 increased from 960 to 4032 [4]. There are no licensed vaccines or specific therapeutics, and substantial vector control efforts have not stopped its rapid emergence and global spread [8].

Dengue fever is still a non-endemic disease in China. No dengue cases were reported in China from 1949 to 1977 until an outbreak occurred in Foshan City, Guangdong Province in 1978, and since then, dengue fever epidemics in China have been intermittent [9]. Dengue fever became a notifiable disease on 1 September 1989 in China, partly in response to the dengue outbreaks reported sequentially in Hainan, Guangxi, Fujian, Zhejiang and Yunnan provinces during the 1980s. All of these provinces are located in the southeastern coastal regions or around the national border with Myanmar, Laos, and Vietnam in Southeast Asia [9]. With the acceleration of globalization and frequent international exchanges, dengue fever in China induced by imported cases has increased substantially [10]. There were 655,324 dengue cases and 610 deaths during 1978–2008, 52,749 dengue cases and six deaths during 2009–2014 and 65,624 cases during 2014–2018 in mainland China [11,12]. In particular, a dengue fever outbreak hit China in 2014, with 47,127 dengue cases in mainland China, 45,231 in Guangdong Province and 37,382 dengue fever cases in Guangzhou City, among which 30,553 cases were clustered in the districts of Baiyun, Liwan, Yuexiu, Haizhu and Tianhe [13]. These dengue outbreaks posed a substantial socio-economic burden in China. Dengue fever outbreaks have spread from Guangdong and Hainan in the southern coastal areas to the relatively northern and western areas including Fujian, Zhejiang, Yunnan, Henan and Shandong, with shorter outbreak intervals compared to those before the 1990s [9,14]. A dengue fever outbreak hit China again in 2019. According to media reports and field prevention and control experiences, China had a much higher infection rate of dengue fever, not only in terms of quantity but also spatial range, in the 2019 outbreak. Although there has been much scientific research on dengue fever characteristics in mainland China before 2019, including both spatio-temporal and crowd features, it is necessary to investigate dengue fever characteristics, especially spatial diffusion, to understand the new spatial dynamics of dengue fever in mainland China. Therefore, this study aimed to explore the spatial characteristics of dengue fever in mainland China in 2019. A better understanding of dengue fever outbreaks will be helpful for determining the allocation of resources for dengue fever prevention and control [15] and can also allow for the planning and implementation of effective public health prevention and control of other epidemics with far higher infection rates and poorer outcomes.

## 2. Methods and Materials

### 2.1. Data Collection

Dengue cases were defined based on laboratory confirmation and clinical diagnosis according to the diagnostic criteria and the principles of management for dengue [16,17].

Dengue fever is a vector-borne notifiable disease in China. Attending physicians are required by law to report to the Chinese Center for Disease Control and Prevention (China CDC) through the Chinese National Notifiable Infectious Disease Reporting Information System (CNNDS). Dengue case reports include each patient’s sex, age, occupation, national code of their current address, date of the illness onset and remarks, etc. Daily dengue case reports from 1 January 2019 to 18 December 2019 were obtained from the CNNDS. Demographic data were obtained from the sixth population census of the National Bureau of Statistics of China for 2010. Data from Chinese administrative divisions, used for geographical mapping, were provided by the CNNDS.

Ethics Statement: No human or animal samples were included in the research presented in this article; therefore, ethical approval was not necessary for this research.

### 2.2. Data Processing

There were a total of 23,647 dengue cases in mainland China in 2019. According to case remarks, dengue cases were divided into indigenous, domestic imported, overseas imported and other dengue cases. Indigenous dengue cases were those patients who did not leave their local counties (their current addresses) within 14 days before illness onset. Domestic imported dengue cases were those patients who left their local counties (their current addresses) and went to other domestic counties where dengue fever was prevalent within 14 days before illness onset. Overseas imported dengue cases were those patients who went to foreign countries or regions where dengue fever was prevalent within 14 days before illness onset. The other dengue cases were those patients that we were not sure how to classify, because the case remarks were blank. According to each case remark, the overseas imported country or the domestic imported province or city or county was confirmed, geocoded and matched to their administrative boundaries (country or province or city or county) for spatial analysis. According to the national codes of current addresses, indigenous dengue cases could be located at the county level and then geocoded and matched to the county-level administrative boundaries. There are 31 provinces (or municipalities) comprised of 2922 counties in mainland China, with populations ranging from 7123 to 5,044,430, with geographic areas ranging from 5.4 to 197,346 square kilometers [18].

### 2.3. Data Analysis

Spatial mapping analysis was performed using ArcGIS version 10.5 (ESRI, Redlands, CA, USA) [19]. The high-risk spatial clusters were explored through Kulldorff’s space–time scan statistic software SaTScan version 9.3 [20]. Purely spatial analysis scanning for clusters with high rates using the Discrete Poisson model was used to determine the average monthly indigenous dengue cases at the county level. Circular scan windows were selected. The maximum spatial cluster size was set as 500 km. Log Likelihood Ratio (LLR) tests were evaluated to decide the significance of identified clusters, and *p*-values were obtained through Monte Carlo simulation after 999 replications. The null hypothesis of a spatial random distribution was rejected when the *p*-value was less than 0.05 [21].

## 3. Results

**Figure 1:**
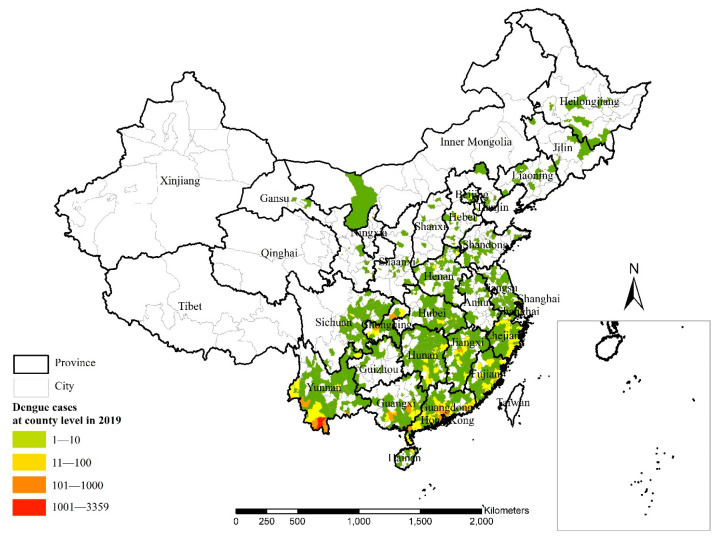
Spatial distribution of dengue cases in mainland China in 2019.

There were 22,246 dengue cases studied in this research. These were distributed in 1211 counties of 28 provinces throughout mainland China, except in for the Xinjiang Uygur Autonomous Region and the Tibet and Qinghai Provinces. Cases included indigenous, domestic imported and overseas imported dengue cases (Figure 1). Most of the dengue cases were distributed in Guangdong (6090 cases) and Yunnan (6818 cases).

### 3.1. Analyses for Indigenous Dengue Cases

**Figure 2:**
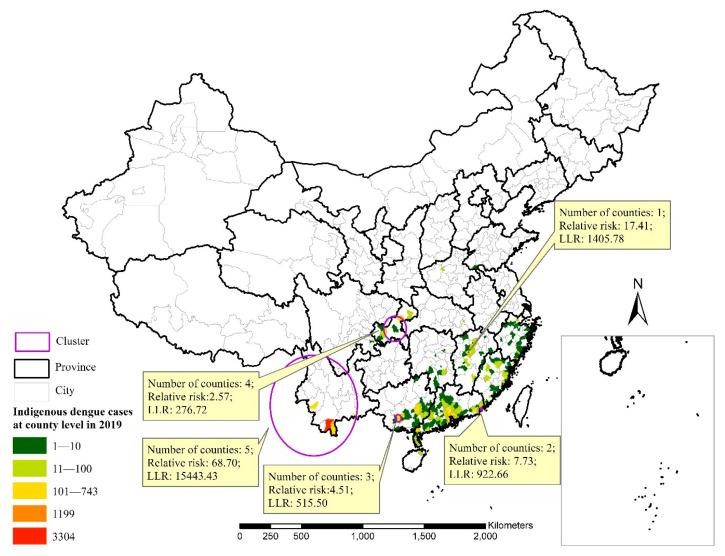
Space–time scan statistical analyses for indigenous dengue cases in mainland China in 2019.

There were 15,187 indigenous dengue cases in mainland China in 2019, distributed in 249 counties of 13 provinces. According to the results of the purely spatial analysis scanning, there were five spatial clusters with LLR values greater than 275.00 (Figure 2). Clusters were found in Ruili City, Dehong Dai Jingpo Autonomous Prefecture and Jinghong City, Xishuangbanna Dai Autonomous Prefecture, Yunnan Province, Zhangshu City, Yichun City, Jiangxi Province, Shantou City, Guangdong Province, Nanning City, Guangxi Province and Chongqing Municipality.

The top six indigenous areas for cases at the county level, accounting for 42.5% of the total number of indigenous cases and colored in red and orange, were Jinghong City, Xishuangbanna Dai Autonomous Prefecture, Yunnan Province (3304 cases); Ruili City, Dehong Dai Jingpo Autonomous Prefecture, Yunnan Province (1109 cases); Zhangshu City, Yichun City, Jiangxi Province (743 cases); Longhu County, Shantou City, Guangdong Province (505 cases); Yubei County, Chongqing Municipality (426 cases) and Jiangnan County, Nanning City, Guangxi Province (364 cases) (Figure 1). The top six areas for indigenous cases at the province level, accounting for 93.8% of the total indigenous cases, were Yunnan Province (5097 cases), Guangdong Province (4497 cases), Guangxi Province (1461 cases), Chongqing Municipality (1158 cases), Jiangxi Province (1071 cases) and Fujian Province (964 cases).

### 3.2. Analysis of Domestic Imported Dengue Cases

#### 3.2.1. Origin Analysis of Domestic Imported Dengue Cases

**Figure 3:**
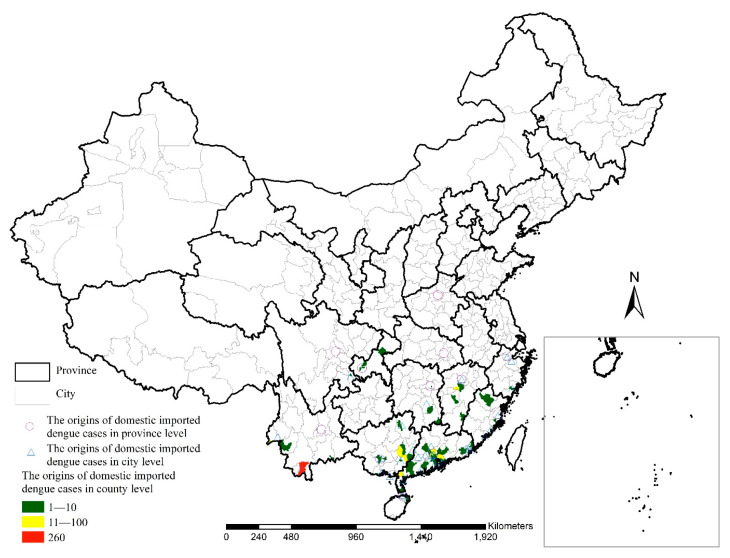
Origins of domestic imported dengue cases in mainland China in 2019.

There were 1281 dengue cases imported from about 80 counties in 12 provinces according to the case remarks. There were 260 cases from Jinghong City, Xishuangbanna Dai Autonomous Prefecture, Yunnan Province (Figure 3). The top two case areas at the city level were Xishuangbanna Dai Autonomous Prefecture, Yunnan Province (390 cases) and Guangzhou City, Guangdong Province (230 cases). The top two case areas at the province level were Guangdong Province (568 cases) and Yunnan Province (447 cases).

#### 3.2.2. Spatial Distribution of Domestic Imported Dengue Cases

**Figure 4:**
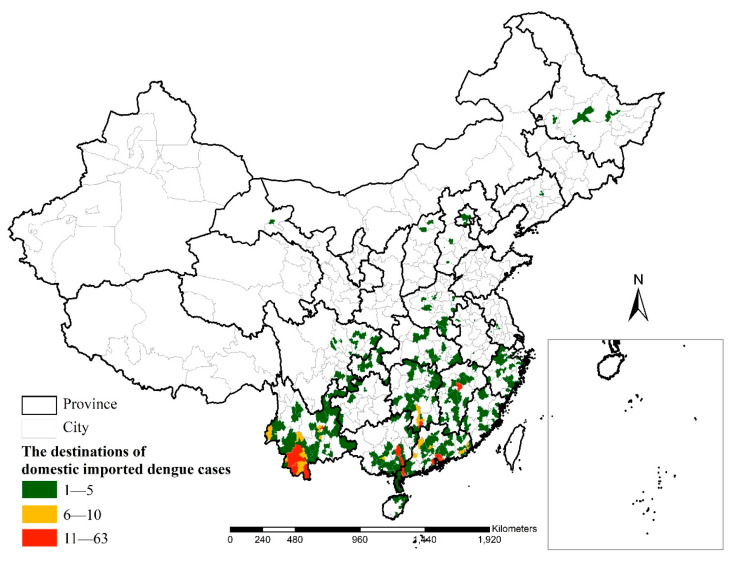
Spatial distribution of domestic imported dengue cases.

There were 1281 domestic imported cases imported into 406 counties of 21 provinces. These cases were clustered in specific counties of Yunnan Province, Guangxi Province, Guangdong Province, Hunan Province and Jiangxi Province. In particular, the top three case areas at the county level were Menghai County, Xishuangbanna Dai Autonomous Prefecture, Yunnan Province (63 cases); Beiliu City, Yulin City, Guangxi Province (49 cases) and Zhongshan City, Guangdong Province (43 cases); these are colored in red (Figure 4). The top four case areas at the province level were Guangdong Province (372 cases), Yunnan Province (348 cases), Guangxi Province (154 cases) and Hunan Province (110 cases), accounting for 76.8% of the total domestic imported cases.

### 3.3. Analysis of Overseas Imported Dengue Cases

#### 3.3.1. Origin Analysis of Overseas Imported Dengue Cases

**Figure 5:**
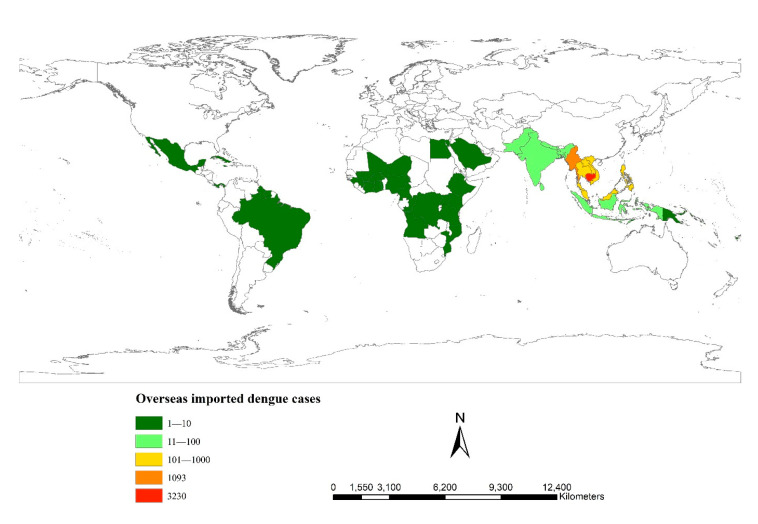
Origins of overseas imported dengue cases in mainland China in 2019.

There were 5778 overseas imported dengue cases from 47 countries in 2019. These cases were mainly from Southeast Asia. Some 1093 cases, 18.9% of the total, were from Myanmar and 3230 cases, 55.9% of the total, were from Cambodia; these are colored in orange and red in Figure 5, respectively.

#### 3.3.2. Spatial Distribution of Overseas Imported Dengue Cases in Mainland China

**Figure 6:**
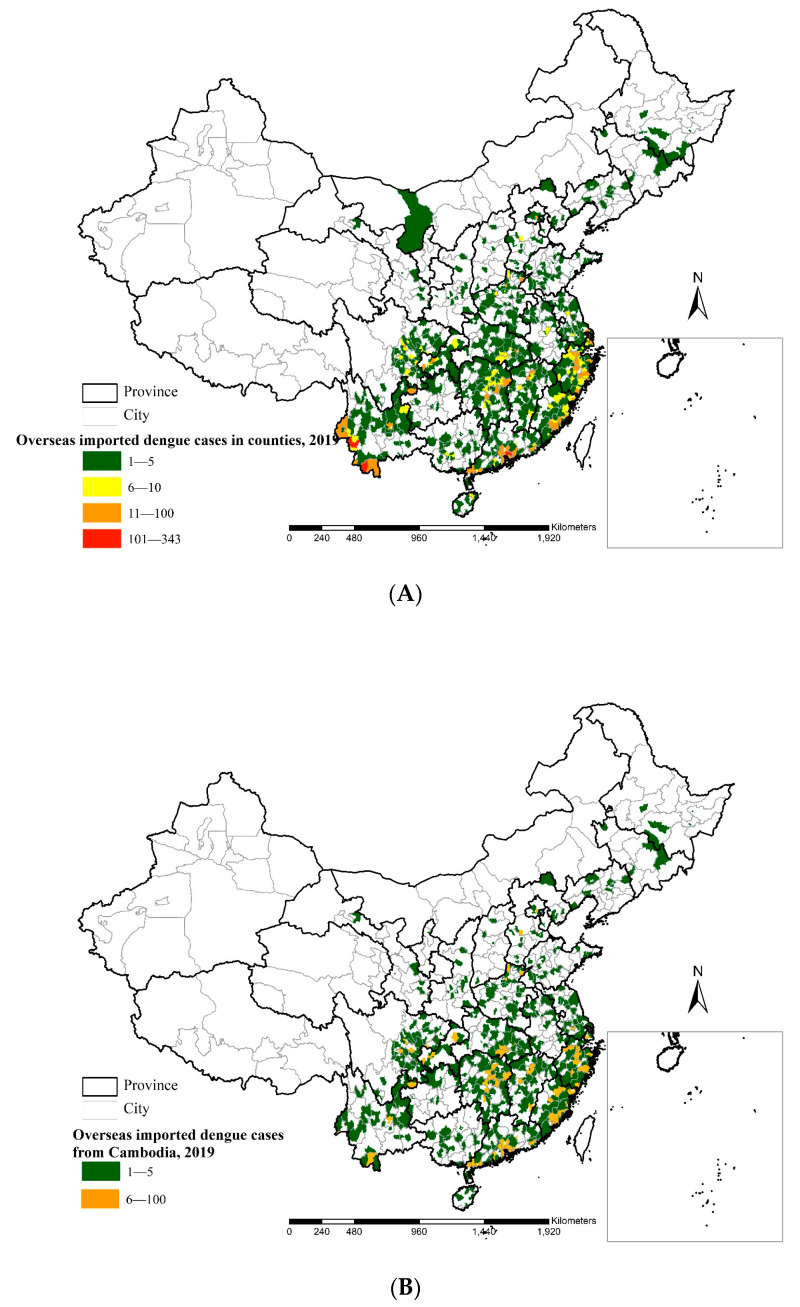

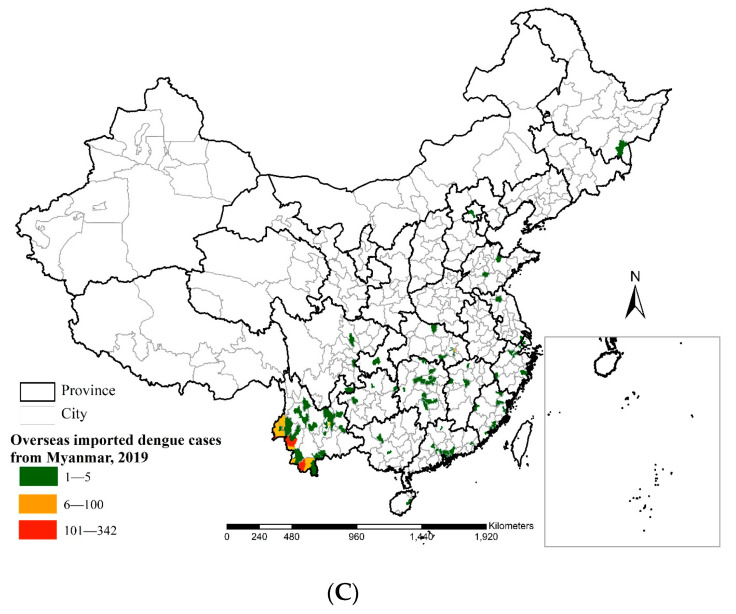
Spatial distribution of overseas imported dengue cases in mainland China in 2019. (**A**). All overseas imported cases. (**B**). Cases from Cambodia. (**C**). Cases from Myanmar.

A total of 5778 overseas imported dengue cases were imported into 1083 counties of 28 provinces throughout mainland China, except for in the Xinjiang Uygur Autonomous Region and the Tibet and Qinghai Provinces. These cases were clustered in the border of Yunnan Province and the coastal areas of Guangdong, Fujian and Zhejiang Provinces, colored in red and orange (Figure 6A). The top two case areas at the province level, accounting for 44.9% of all cases, were Yunnan Province (1371 cases) and Guangdong Province (1221 cases).

A total of 3230 overseas imported dengue cases from Cambodia were imported into 908 counties of 27 provinces located in the eastern regions along the southwest-northeast line in mainland China (Figure 6B). The top three case areas at the province level, accounting for 50.3% of all cases, were Guangdong Province (823 cases), Fujian Province (464 cases) and Zhejiang Province (338 cases).

A total of 1093 overseas imported dengue cases from Myanmar were imported into 136 counties of 21 provinces (Figure 6C). Most of the cases were clustered in the border region of Yunnan Province. There were 971 cases in Yunnan Province, accounting for 88.8% of all cases.

## 4. Discussion

**Figure 7:**
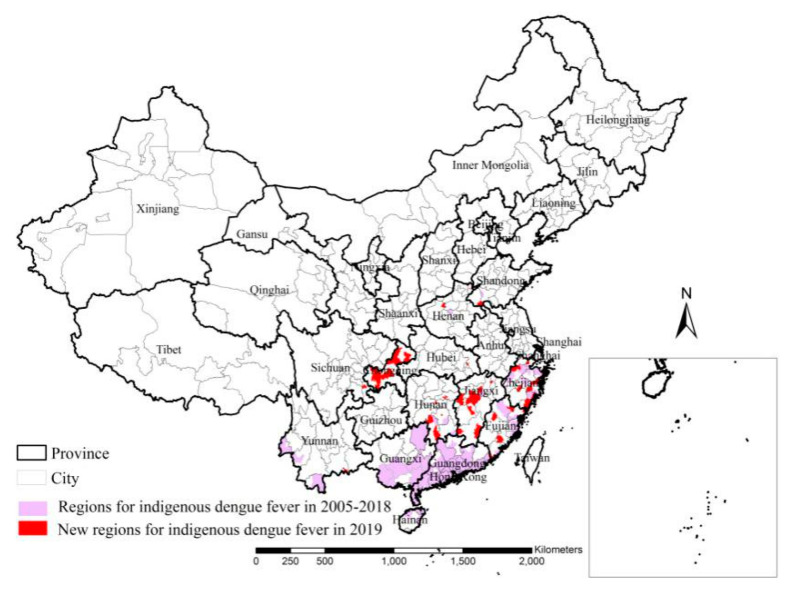
Comparative analysis of indigenous dengue fever cases between 2019 and 2005–2018.

There were 19,508 indigenous dengue cases in mainland China during the period 2005–2018, except for in 2014, and most of the cases were distributed in the Guangdong (57.1%) and Yunnan (28.6%) Provinces (Table 1). An extensive dengue fever outbreak, which posed a substantial socio-economic burden, hit China in 2014 [11], and 97.3% of the indigenous dengue cases occurred in Guangdong Province. There were 15,187 indigenous dengue cases in mainland China in 2019, much more than those for every year during the period 2005–2018, except for 2014. Dengue fever first occurred in 2005 in 76 counties of 10 provinces and it occurred initially in three provinces, Sichuan and Hubei Provinces and Chongqing Municipality, in 2019 (Figure 7). In total, 17, 75 and 1158 cases occurred in these three provinces, respectively. The previous studies also showed that more than 90.0% of indigenous cases were located in Guangdong and Yunnan Provinces during the period 2005–2018 [12,22,23]. However, only 63.2% of indigenous dengue cases were located in Guangdong and Yunnan Provinces in 2019, and there were also many indigenous cases in Guangxi Province, Chongqing Municipality, Jiangxi Province and Fujian Province, accounting for 30.6% of the total. Therefore, dengue fever was very prevalent and widely distributed in several provinces in 2019 besides Guangdong and Yunnan Provinces. Recognizing the dynamics of indigenous dengue fever in China is important for the development of efficient prevention and control strategies.

According to previous studies, 872 domestic imported cases from eight provinces were imported into 267 counties of 20 provinces in mainland China from 2014 to 2018 [24]. However, 1281 domestic imported cases from 12 provinces were imported into 406 counties of 21 provinces in mainland China in 2019. Domestically imported cases were mainly from Guangdong Province from 2014 to 2018 [24], but mainly from Yunnan and Guangdong Provinces in 2019. Guangdong Province, located in the southeastern coastal region, with a large number of overseas imported and indigenous cases, a developed economy and a large transient population, is conducive to the export of cases. Yunnan Province, located in the southwest border, with many overseas imported and indigenous cases, an underdeveloped economy and many migrant workers, is also conducive to the export of cases. Therefore, domestic imported dengue fever was more prevalent and widely distributed in terms of both case quantity and spatial range in 2019. The global incidence of dengue fever has grown dramatically in recent decades, and about half of the world’s population is now at risk [4]. China has a large population of 1.4 billion and also faces huge population migration. Consequently, dengue fever control and prevention in China might help prevent a global increase or outbreak. It is very important to inform people about dengue fever and improve the awareness of dengue prevention and control.

**Figure 8:**
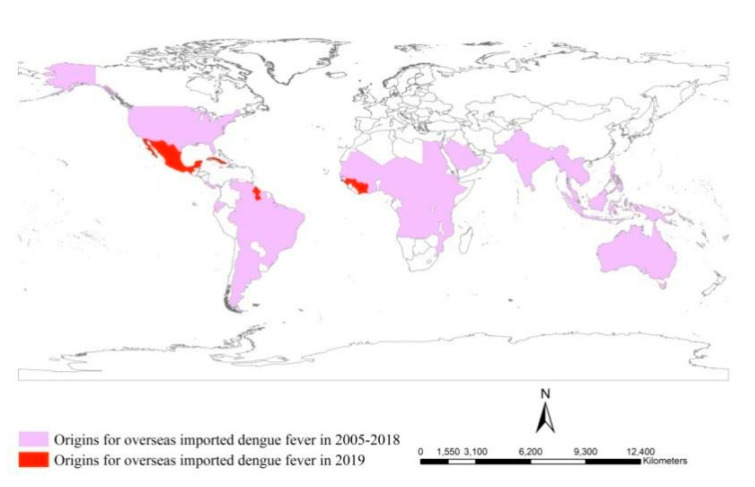
Comparative analysis of overseas imported dengue cases between 2005–2018 and 2019.

Imported dengue cases were from 66 countries located in Africa (26 countries), Asia (18 countries), North America (eight countries), South America (seven countries) and Oceania (seven countries) during the period 2005–2019 (Figure 8) [22,24]. In 2019, there were five new sources of importation, namely Cuba, Guyana, Guinea, Ivory Coast and Mexico. There were 6654 overseas imported dengue cases during the period 2005–2018 [12,22]. However, there were 5778 overseas imported dengue cases in 2019. Although a dengue fever outbreak, with 47,127 dengue cases, hit mainland China in 2014, only 399 were overseas imported dengue cases [24]. Thus, overseas imported dengue fever was very prevalent in 2019. More than 50.0% of the overseas imported dengue cases were from Myanmar, close to the Yunnan border of China during the period 2014–2018, which were mainly imported into Yunnan Province [12]. However, 55.9% of overseas imported dengue cases were from Cambodia in 2019, most of which were imported into Guangdong, Fujian and Zhejiang Provinces. Of the total, 18.9% were from Myanmar in 2019, most of which were imported into Yunnan Province. In 2019, Chinese businessmen set up new factories in Cambodia and organized a large number of domestic workers to work there, which resulted in an increase of imported dengue cases from Cambodia. Therefore, we should be alert to any change of the origins and imported destinations of overseas imported cases and make adjustments in the management of overseas imported dengue fever such as the strengthening of epidemiological investigations, monitoring of body temperature and isolating of imported patients.

A total of 79.1% of indigenous cases in mainland China in 2019 were located in Yunnan, Guangdong, Guangxi and Fujian Provinces, all belonging to the southwestern border and southeastern coastal regions. Further, 14.7% of those were located in Chongqing Municipality and Jiangxi Province, both belonging to the central regions. Yearly rainfall in these regions ranges from 800 mm to 1600 mm or even greater than 1600 mm and belong to the torrid and sub-torrid zones [25]. Indigenous dengue fever also occurred in the regions belonging to the warm temperate zone with yearly rainfall of 400–800 mm, such as Henan and Shandong Provinces. *Aedes aegypti* and *Aedes albopictus*, the vectors of dengue fever, are distributed in the outer areas along the southwest-northeast line of China, which are much larger than the regions with indigenous dengue fever [26]. The hatching and growth of *Aedes aegypti* and *Aedes albopictus* are directly affected by weather conditions such as temperature and rainfall [27]. Furthermore, dengue fever is closely related to the economy, traffic, population mobility, climate warming, *Aedes* mosquito density and overseas imported dengue fever, etc. [28,29,30,31]. Considering the current spatial distribution of dengue fever and mosquito vectors, climate and environmental factors, large population density and population migration, dengue fever is likely to spread further in mainland China in the near future, and we should improve the risk awareness of dengue fever and actively take measures to prevent it.

## 5. Conclusions

Dengue fever was very prevalent and widely distributed in mainland China in 2019. There were many more dengue cases than those for every year during the period 2005–2018, except for in 2014. There were also some big changes in the spatial distribution and proportion of dengue fever. In particular, indigenous dengue fever in 2019 occurred in new regions such as Sichuan Province, Chongqing Municipality and Hubei Province. The mastering of new spatial characteristics of dengue fever in China is helpful for its prevention and control and for assessing and dealing with its risks.

## Figures and Tables

**Table 1:** Comparative analysis of indigenous dengue cases between 2019 and 2005–2018.

Province	Indigenous Dengue Cases		
	During 2005–2018 Except 2014	in 2014	in 2019
Zhejiang	1447	2	306
Anhui	1		
Fujian	1116	253	964
Jiangxi	1		1071
Shandong	79		2
Henan	29		75
Hubei			24
Hunan	98		262
Guangdong	11,130	44,795	4497
Guangxi	17	841	1461
Hainan	12	2	253
Chongqing			1158
Sichuan			17
Yunnan	5578	138	5097
Total	19,508	46,031	15,187

## Data Availability

Dengue fever reports and the vector data of Chinese administrativedivisions were available from Chinese National Notifiable Infectious Disease Reporting InformationSystem (http://www.chinacdc.cn/ (accessed on 31 December 2020)). Demographic data was obtained from the sixth population census of the National Bureau of Statistics of China (http://www.stats.gov.cn/ (accessed on 31 December 2020)).
